# Nonfacial Skin Rejuvenation of the Neck, Chest, and Hands. Part Two: Using Laser Techniques

**DOI:** 10.1111/jocd.16671

**Published:** 2024-11-15

**Authors:** Callie Fares, William Abou Shahla, Mariana El Hawa, Dana Saade

**Affiliations:** ^1^ Department of Dermatology American University of Beirut Medical Center Beirut Lebanon

**Keywords:** fillers, injections, lasers, nonfacial, rejuvenation, skin

## Abstract

**Background:**

Interest in aesthetic procedures that help maintain a youthful look is on the rise. While many nonsurgical techniques focus on facial rejuvenation, there is a need for a detailed review of treatments, specifically for nonfacial areas.

**Aim:**

This review explores various clinic‐based methods for revitalizing the neck, chest, and hands, with a particular emphasis on different laser treatments.

**Methods:**

For this second part of the review, we conducted a comprehensive search on PubMed to evaluate the effectiveness of various laser treatments. The review covers treatment parameters such as wavelength, fluence, and pulse, along with outcomes, follow‐up care, and potential side effects. A discussion on the role of sclerotherapy in treating these areas was also included.

**Conclusions:**

This article compiles recent findings on the safety and effectiveness of these treatments, showcasing progress in laser technologies and the growing trend of noninvasive body rejuvenation. It offers practical insights for both practitioners and patients looking for reliable options in nonsurgical body treatments.

## 1 Introduction

This is part 2 of a two‐part series review on skin rejuvenation of nonfacial body parts, which predominantly concentrates on various types of lasers, providing specific details on parameters, follow‐up times, and some potential side effects. The first part focused on various types of fillers, showing their preparation, the required volume per specific area, the injection methodology, durability, and if present, any associated side effects. The use of some other procedures such as mesotherapy, microneedling, chemical peeling, and Profhilo in such body areas was also discussed.

## 2 Lasers

### 2.1 Intense Pulsed Light (IPL)

To address aging, several nonablative skin resurfacing methods are available for facial rejuvenation, with intense pulsed light (IPL) being particularly effective (Table 1). IPL uses polychromatic, noncoherent, and noncollimated flashlamps to target specific chromophores depending on the wavelength. This selective targeting makes IPL a flexible treatment option for various purposes, including hair removal, treating pigmented or vascular lesions, and enhancing skin appearance [1].

For skin rejuvenation purposes, IPL can be used to diminish the appearance of pigmented spots for a more homogeneous look. Therefore, the IPL targets pigmented lesions caused by melanin, using a wavelength of 630–1100 nm [2]. For vascular lesions, cutoff wavelengths are used to target hemoglobin, at a wavelength of 515–590 nm [3].

IPL has been shown to modulate the production of neocollagen in treated areas, in the papillary and reticular dermis. It has also been found that lower concentrations of metalloproteinases 1 and 2 are found, leading to less destruction of collagen in the extracellular matrix [3].

IPL treatment should not be performed on patients with darker skin tones (types V to VI), moles, and other pigmented spots, due to the risk of hyper/hypopigmentation and scarring [4]. Patients with autoimmune diseases, like psoriasis and vitiligo, should not undergo IPL treatment for increased risk of the Koebner phenomenon. Patients with the history of epilepsy, herpes simplex virus (HSV), keloid scarring, superficial cuts, or the use of photosensitizing medications (e.g., minocycline and isotretinoin) should also avoid IPL [4]. Erythema is a common side effect post‐procedure but can disappear hours to 3 days later [3].

#### 2.1.1 Neck

IPL can be used for the treatment of poikiloderma of Civatte and photorejuvenation of the neck. When comparing the face, neck, and chest for rejuvenation and photoaging improvement using IPL, the face shows the most improvement followed by the chest and then the neck [5]. To target melanin and hemoglobin absorption, a cutoff of 515 nm is used for the treatment of pigmentation and telangiectasias [6]. Rusciani et al. performed IPL therapy on 175 patients on the chest and neck to determine the efficacy and adverse effects on the poikiloderma of Civatte. Different filters were used, including 515, 550, and 590 nm, to target the vascular and pigmented components of superficial and deep tissues [7].

#### 2.1.2 Chest

The chest is evaluated to determine the Fitzpatrick skin type, the presence of hyperpigmentation, vascular structures, actinic keratosis, fine lines, wrinkles, laxity, and erythema [8]. With the use of Lumenis 1 or M22, Peterson and Goldman used the multiple sequential pulsing technique to let the skin cool between pulses; this technique involves 10–30 and 30–40 ms delays between skin types I to III and skin type IV, respectively.

The face responds better compared to the chest and neck; therefore, more treatments may be needed for the chest and neck [5]. No specific adverse effects have been seen on the chest when compared to the face.

#### 2.1.3 Hands

Due to IPL's effectiveness with minimal downtime and side effects, it has been studied for removing solar lentigines and wrinkles on the dorsal hand. The use of IPL on the hands has also shown to improve skin texture, like in the chest and neck. Maryuma studied the use of IPL on the hands on 128 patients. A wavelength of 560 nm was used to target wrinkles, senile lentigines, and diffuse melanin, while a 515 nm wavelength targeted melanin. After one to two treatments, improvement in dorsal senile lentigines and wrinkles was seen in 59% and 25% of cases, respectively [9]

**TABLE 1:** IPL parameters, adverse events, and post‐procedure care on the neck, chest, and hands.

Laser type	Mechanism of action	FDA approval	Parameters			Follow‐up session	Results	Side effects
			Wavelength (nm)	fluence (J/cm^2^)	Pulse (ms)			
IPL	Works by targeting specific chromophores (cellular or structural elements) in the epidermis or dermis. The targeted chromophores are damaged at cellular, or larger tissue, levels by photothermolysis [2]	Yes [6]						
Neck			Cutoff of 515 [7], 550, and 590 nm [7] Longer wavelengths resulted in better improvement in pigmentation; superficial vessels were targeted better with shorter wavelengths [7]	32–36 J/cm^2^ [7]	Double or triple pulse with 2.5–3.5 ms pulse duration 10–20 ms pulse delay [7]	Three treatments at 3–4 weeks intervals [7]	Marked reduction in poikiloderma lesions in 82% of their patients	Erythema, swellings, microcrust formation, blisters [7] mild purpura, and hypopigmentation
Chest			Skin types I–III: Cutoff of 560 nm Skin type IV: Cutoff of 590 nm [3]	18–22 J/cm^2^ [3]	Double pulse with 3 ms pulse duration (predominant hyperpigmentation), 3.5 ms pulse duration (prevalent erythema), or 4 ms pulse duration (predominantly consists of fine telangiectasia) Multiple sequential pulsing technique: 10–30 ms delay (skin types I–III) or 30–40 ms delay (skin type IV) [3]	3–4 treatments every 4 weeks [8, 10]	Has the benefit of being a short session, typically 5–10 min for each treatment of the chest; healing time is less than 3 days after each session [10] Some studies report lasting results for up to 4 years [3]. Improvement of rhytides, telangiectasia, and other appearances of photoaging can also be seen up to 45 days post‐treatment [10]	Same as above
Hands			Wrinkles, senile lentigines, and diffuse: 560 nm or Melanin: 515 nm [9]	12 J/cm^2^ or 13 J/cm^2^ [9]	Double pulse with 4.0 ms pulse duration, 30 millisecond delay or A single pulse, 3.0 ms pulse duration [9]	1–6 treatments, 1 month apart [9]		Erythema, post‐inflammatory hyperpigmentation, itching, blister formation, and scar Crusting on heavily pigmented lesions which resolves in 2 weeks [9]

### 2.2 Photodynamic Therapy (PDT)

Photodynamic therapy (PDT) is the use of a photosensitizing drug and its activating wavelength of light to achieve the destruction of target tissue, through a photochemical reaction involving oxygen [11]. When the drug 5‐aminolevulinic acid (ALA) or methyl aminolevulinate (MAL) is applied topically, PDT has shown to improve skin roughness, fine lines, complexion, skin smoothness, and reduce actinic elastosis [12]. PDT is also used in the removal of actinic keratosis and is approved by the FDA [11]. Irrespective of the light source used, whether an incoherent lamp or an LED system, Babilas et al. found that in a split face study after one session using topical ALA‐PDT, the appearance of wrinkles and hyper‐/hypopigmentation improved after 3 months [13]. PDT is not the treatment of choice of deep wrinkles, but rather more invasive techniques should be used [12].

When performing PDT, the skin should be cleaned before applying ALA, using either acetone, soaps, isopropyl alcohol, alpha‐hydroxy or salicyclic acid cleansers, or towels. Next, the stratum corneum is disrupted in order to allow efficient penetration by ALA, which can be done by gauze abrasion or microneedle rollers. ALA is then applied to the area to be treated, and for areas outside the face, an occlusive‐like plastic wrap should be used, which will allow for the increase of ALA infiltration into the stratum corneum [11]. The incubation period varies with the PDT use but is generally 30 min to 3 h for skin rejuvenation when using ALA. It should be done in a dark or lightly lit room, in order to prevent damaging of the skin due to UV penetration [11, 14]. This incubation time increases by 15–30 min for each of the subsequent treatments [14]. The light source is chosen according to the photosensitizer present in the skin [15]. Two to three sessions, with at least 4 weeks in between, are needed to treat signs of photoaging using PDT [12].

Adverse effects include pain, erythema, edema, blistering, phototoxicity, infection, immunosuppression, scarring, and pigmentation [11, 12, 15]. Pain during treatment is expected and may last up to 48 h. Pain is more tolerable if actinic keratoses are not present, and PDT is only being used for photodamaged skin. It is possible to take breaks and interrupt the irradiation process to decrease the pain; however, the length of the treatment will be increased. Cooling techniques, like thermal water or gel, cold cream, or cool pads, are recommended following treatment [12]. Sun exposure and use of corticosteroids should be strictly avoided for weeks after therapy [11, 12]. Erythema is more prolonged in the chest area compared to the face (Table 2).

**TABLE 2:** PDT parameters, adverse events, and post‐procedure care on the neck, chest, and hands.

Laser type	FDA approval	Parameters			Follow‐up session	Side effects
		Wavelength (nm)	Fluence (J/cm^2^)	Pulse (ms)		
PDT	No [19]					
Neck		5% ALA cream (2 h of incubation), red LED 630 nm [20]	60 J/cm^2^ [20]			Pain, erythema, edema, and hyperpigmentation which were all transient [20]
Chest		ALA (incubation 30 min), 417 nm (blue light) [21] or PDL, IPL, red light, or blue light [3]	10 J/ cm^2^ [21]	1000 s [21]		Erythema takes longer to heal and to re‐epithelialize, leading to longer persistent erythema following PDT [12]
Hands		PDL, IPL, red light, blue light, or daylight [17] 20% aminolevulinic acid solution or a 16% methyl aminolevulinate cream, 595 nm PDL [16]	10–12 J/cm^2^ [16]	40 ms pulse width, 7 mm spot size [16]	1 Treatment [16]	Pruritus, erosions, pain, scaling, and crusting [16]

#### 2.2.1 Neck

The neck is more sensitive to pretreatment methods, due to its slower re‐epithelialization compared to the face; however, the method of PDT performed is as described above [12]. Zhang et al. compared the results of not using a photosensitizing drug versus ALA‐PDT using IPL and red light on photodamaged neck skin. Their results showed better rejuvenation using ALA‐PDT when compared to no photosensitizer, by showing improvement in skin appearance, hydration, transepidermal water loss, thickness, and elasticity. Post treatment, erythema is prolonged on the neck [12].

#### 2.2.2 Chest

When choosing what pretreatment method to use, it should be taken into consideration that the skin of the chest differs from that of the face, having less sebaceous glands and appendages. The chest therefore takes longer to heal and to re‐epithelialize, leading to longer persistent erythema following PDT [12]. Treatment parameters can be seen in Table 2.

#### 2.2.3 Hands

PDT has been extensively studied for treating actinic keratosis on the hands, with the added benefit of improving signs of photoaging, such as fine lines, mottled hyperpigmentation, sallowness, and uneven texture [16, 17]. Martin has reported greater enhancement of skin appearance on the dorsal hand after ALA‐PDT, when combined with 0.5% 5‐fluorouracil cream [18]. Fabi et al. recommend one session for photorejuvenation and two to three sessions for the treatment of actinic keratosis. ALA or MAL is placed on the dorsal hand for 1 h after performing microdermabrasion, followed by activating the photosensitizer with a series of PDL, IPL, blue light, and red light. The pretreatment practice can be performed more aggressively when compared to the neck and chest; healing of the hands takes longer than the face, and the session is less painful [12].

### 2.3 Nonablative Fractional Lasers (NFL)

Nonablative lasers (NFL) resurface the skin without damaging the epidermis [22]. The improvement in skin tones and textures is accomplished by heating deeper layers to stimulate collagen production [23, 24, 25]. Typically, using wavelengths between 1320 and 1927 nm, they offer less recovery time and fewer side effects than ablative lasers, though they are less invasive and less effective [23].

The most studied nonablative laser is the fractionated 1550 nm (a water‐absorbing, mid‐infrared wavelength) erbium‐glass laser (Table 3) [3, 22, 26]. The laser is fractionated in order to produce microscopic, coagulated tissue columns, termed microthermal treatment zones (MZT), leaving untreated epidermis around the MZTs [24, 26]. MZTs penetrate the skin up to 1600 μm in depth, with a 100–200 μm diameter [26]. The pattern of coagulated tissue and the surrounding untreated epidermis/adnexal structures allow for faster healing of the skin [26, 27]. Therefore, nonablative fractional lasers can be used on areas other than the face, including the trunk and extremities, which have reduced ability to re‐epithelialize due to decreased pilosebaceous glands [26].

**TABLE 3:** NFL parameters, adverse events, and post‐procedure care on the neck, chest, and hands.

Laser type	FDA approval	Parameters			Follow‐up session	Side effects
		Wavelength, (nm)	Fluence (J/cm^2^)	Pulse (ms)		
NFL	Yes					
Neck		1540 nm erbium‐glass fiber laser [31]	15 mJ/microbeam [31]		Six sessions, every 3–4 weeks [31]	Edema (1–3 h), erythema (1–3 days), and fine scaling (in four patients only and lasted less than a week) [31]
Chest		1540 nm erbium‐doped laser treatment [28]	10–40 mJ [3]	Eight passes [3]		Erythema [28]
Hands		1550 nm erbium‐diode fiber laser [33]	6–12 mJ [33] or 8 to 9 mJ/MTZ [34]	Total treatment densities of 1000–2000 MTZ/cm^2^ [33] or Ten passes at 250 MTZ/cm^2^ (final treatment density of 2500 MTZ/cm^2^) [34]	Five to six treatment sessions, every 2–3 weeks [33]	Pain linked to the settings used, erythema, and edema (resolved within a month) [33]

NFL is safe for all Fitzpatrick skin types, as it targets water in the cells rather than melanin, making it suitable for darker skin without causing pigment changes. While pretreatment with hydroquinone has been recommended for skin types III and IV, data supporting its effectiveness are limited [22, 25]. Uses of NFL include treating scars (acne, surgical, and traumatic), photodamage‐associated dyschromia, actinic keratosis, melasma, striae distensae, skin laxity, and wrinkles [22, 24, 26]. However, it has been shown that improvement of skin laxity and rhytides is minimal with NFL [22, 26].

The skin is prepared for treatment first by cleaning and then applying a topical anesthetic for 1 h [28]. The procedure takes between 30 min and 2 h depending on the size of the treated area [23]. Intervals between sessions vary depending on the desired results; patients should wait between 2 and six weeks until the next treatment [29]. Sun exposure should be avoided both pre‐ and posttreatment in order to minimize erythema and other adverse effects [28]. Patients should wait until their tans fade and should use broad‐spectrum sunscreens during the course of the treatment [25].

Pain during treatment is common and can be minimized with local anesthetics and cold air cooling [3]. In general, NFL is well tolerated [26]. Adverse effects reported in the literature due to NFL include blisters, prolonged erythema, scarring, petechiae, pinpoint hemorrhaging, recurrent melasma and post‐inflammatory hyperpigmentation, acneiform eruptions, prolonged edema, dermatitis, impetigo, purpura, HSV outbreak, and erosions [3, 22, 28]. Darker skin types were correlated with more adverse effects [28]. Indications to account for pretreatment include holding retinoid and isotretinoin use due to decreased wound healing; pregnancy and breastfeeding due to increased risk of pain and discomfort during the procedure and hyperpigmentation; and history of silicone injections due to prior reports of developing lumps in the area treated [22, 30]. Although NFL is relatively tolerable and has minimal recovery time, results related to rejuvenation are much less effective than other preferred treatments [26, 29].

#### 2.3.1 Neck

Bencini et al. treated dyschromia, skin laxity, and wrinkles on the neck of 18 females using a 1540 nm erbium‐glass fiber laser. Patients underwent six sessions spaced 4 weeks apart, with an energy of 15 mJ/microbeam and a density of 3600/4000 microthermal zones/cm^2^. Significant improvement in dyschromia and wrinkles was observed, but no improvement in skin laxity, which Bencini et al. attributed to the laser's limited penetration depth [31, 32]. Wanner et al. evaluated the effectiveness of NFL on facial and nonfacial areas for improving photodamage, rhytides, and dyspigmentation. Three treatment sessions were performed on the neck and chest at 3–4 week intervals. The best results were observed 3 months post‐treatment, with improvements diminishing at 6 and 9 months, likely due to UV exposure or reduced epidermal effect. The authors noted that conservative parameters used on nonfacial areas led to less improvement compared to the face, and they recommend using higher energy settings in future treatments for better outcomes in photodamage, rhytides, and dyspigmentation [32]. This method is a safer alternative to ablative techniques and may be preferred by some patients.

#### 2.3.2 Chest

NFL was initially used solely for face rejuvenation but has been shown to benefit signs of mild‐to‐severe photoaging on the chest [3]. Erythema lasts longer on the chest than the face [28]. When treating the skin of the chest, it has been suggested to use eight passes with fluences of 10–40 mJ. Darker Fitzpatrick skin types (IV–VI) need lower treatment levels of 4–7 when compared to skin types I–III, the latter treating up to levels 7–11 [3].

#### 2.3.3 Hands

NFL causes less skin damage compared to other laser treatments, making it a safer option for rejuvenating nonfacial areas like the hands. Its benefits include visible improvement with minimal downtime, though further research is needed to confirm its superiority over ablative techniques. In a study by Sadick et al. on nine individuals with moderate bilateral photodamage on the dorsal hands, a 1550 nm erbium‐diode fiber laser was used. After 1 and 3 months, both investigators and patients noted improvements in wrinkles, skin texture, and pigmentation. Histological samples from three individuals were analyzed 6 months post‐treatment to link improvements with changes in collagen and elastin composition. Patients underwent five to six sessions, spaced 21–28 days apart. Overall, the results were positive, showing improvements in photodamage, rhytides, pigmentation, and surface texture [31, 33]. Histological samples showed improvements in photoaging signs, including higher collagen density, fewer cellular abnormalities (e.g., atypical keratinocytes), enhanced rete ridges, and healthier collagen [33]. Jih et al. showed similar improvement in the signs of photoaging of the hands after five treatment sessions with a 3‐week interval—with particular improvement of dyschromia surpassing to improvement in skin roughness and wrinkling [34]. Safe parameters used for the hands include energies of 6–12 mJ and total treatment densities of 1000–2500 MTZ/cm^2^ [33, 34]. Although improvement has been shown with minimum downtime and manageable adverse effects, there are mixed results when using NFL on the hands due to many treatment sessions needed when compared to ablative treatments [33, 35].

### 2.4 Ablative Fractionated Lasers (AFL)

As opposed to nonablative lasers, ablative lasers destroy the epidermis while heating the dermis in order to stimulate new collagen formation by targeting water molecules via photothermolysis [23, 36]. Light is transformed into heat, which promotes regeneration, repair, and tissue remodeling due to the thermal damage done by the laser to achieve the desired results [36]. Results may not be seen directly; it has been found that neocollagenensis can occur for up to 6 months. Therefore, it may take up to 1 year to be able to appreciate the final result of treatment [35].

The prototype AFL is the CO_2_ laser (Table 4). It emits infrared radiation at a wavelength of 10 600 nm, and its target chromophore is water. Different depths can be reached for different indications (superficial skin condition vs. deep scar remodeling) by increasing or decreasing pulse energies, with a higher energy penetrating deeper [37, 38]. Deep fractionated CO_2_ lasers cause reduced epithelial damage to phototype IV and darker, leading to reduced risk of hyperpigmentation [37, 39, 40].

**TABLE 4:** AFL parameters, adverse events, and post‐procedure care on the neck, chest, and hands.

Laser type	FDA approval	Parameters			Procedural notes	Follow‐up session	Side effects	Avoid
		Wavelength, (nm)	Fluence (J/cm^2^)	Pulse (ms)				
AFL	Yes [47]							
Neck		10 600 nm CO_2_ laser [29, 45]	20 W [29, 45], less than 30% density [43, 44]	500 ms [29, 45]	Extra care must be taken to prevent adverse effects [44] Local anesthetics are applied to the skin at least 30 min before treatment [29, 36, 40]. During treatment, cool air is simultaneously applied for additional relief [29, 40]. Post‐treatment care includes avoiding sun exposure for at least 4 days, using sunscreen on the treated area, applying a cold compress to the treated area for swelling, washing gently (starting 24 h after treatment), and applying healing ointment or cream as indicated [40, 46].	One to three treatments, every 6–8 weeks [29, 45]	Erythema and edema, pruritus [29, 45], scarring that is complicated with infection [43, 44] Precautions should be taken when treating the neck, by decreasing the energy and/or density (less than 30%) compared to the face [43, 44]. Some doctors advise to use prophylactic antibiotics when treating large areas and to closely follow‐up patients post‐treatment [43]	
Chest		10 600 nm CO_2_ laser [44]	10 mJ [44], 120 mm spot and no greater than 5 or 10% density [44]	[44]	Local anesthetics are applied to the skin at least 30 min before treatment During treatment, cool air is simultaneously being applied for additional relief [29, 40] Post‐treatment care includes avoiding sun exposure for at least 4 days, using sunscreen on the treated area, applying a cold compress to the treated area for swelling, washing gently (starting 24 h after treatment), and applying healing ointment or cream as indicated [40, 46].	4–12 weeks interval in between [46]	Erythema, edema, PIH, pain, peeling, bleeding, burning sensation, crusting, scaling risk of infection, contact dermatitis, acneiform eruptions [36, 38, 40, 42, 46]	Keloids, vasculitis, radiodermatitis, sensitivity to light, immunosuppression, ongoing skin or systemic infection, surgical or aesthetic intervention at the treatment site, conditions with reduced adnexal structures (e.g., post‐radiation therapy or scleroderma), diseases prone to the Koebner phenomenon (e.g., psoriasis and vitiligo), and previous isotretinoin use [30, 48]
Hands		10 600 nm CO_2_ laser [35]	20 W, 500 μm spacing [35]	500 or 700 ms pulse duration [35]		Two to three treatment sessions [35]	Mild residual erythema [35]	

The Er:YAG laser (erbium‐doped yttrium aluminum garnet laser) has a wavelength of 2940 nm, targets water as well, and can be used for photodamaged skin and scarring [41]. Er:YAG can be used in darker skinned individuals with phototypes III–V and is often the preferred treatment modality [36, 41]. The melanin in phototypes IV–VI may be prone to pigmentary alterations caused by melanosome rupture, but this was only seen in one of eleven studies reviewed by Modena et al. It is used to treat deep rhytides, photodamaged skin, burns, traumatic scars, unifying skin tone, skin spots, and acne [36, 37, 40, 42]. When the CO_2_ and Er:YAG lasers are compared, their efficacy and complications are relatively the same. In addition, both lasers have a comparatively similar cost, and there are no studies proving the use of one laser over the other [39]. Therefore, it is up to the availability of the lasers in the clinic to decide which device to use.

Theoretically, the use of ablative lasers is the ideal treatment for skin rejuvenation. However, due to the many adverse effects (oozing and crusting, erythema, infection, and scarring) and increased healing time of full ablation, the solution for a more efficient treatment was found by fractionating the laser [37]. Just like in NFL, this creates MZTs in between untreated areas allowed for lower post‐treatment downtime, a safer treatment spectrum, and less adverse effects when compared to full ablative lasers [36, 37, 40, 43]. Re‐epithelialization and the formation of new collagen using fractionated CO_2_ can begin as soon as 48 h post‐treatment, due to the undamaged areas allowing for an earlier healing initiation [38, 43].

#### 2.4.1 Neck

Full ablative lasers were contraindicated for areas other than the face, due to decreased pilosebaceous glands and a thinner dermis causing compromised healing [35, 37, 44, 45]. However, with the appropriate conservative settings, AFL can be used for areas other than the face, including the neck, chest, arms, hands, back, and abdomen [35, 37, 40]. The neck is a very sensitive area to treat, and extra care must be taken to prevent adverse effects [44].

Tierney et al. demonstrated the use of ablative fractional CO_2_ lasers on the neck of 10 different patients to determine the efficacy in improving the appearance of skin laxity, texture, and rhytides. Based on the scoring system, all patients improved in all three cosmetic outcomes—skin laxity, texture, and rhytides—by at least one point out of five, with the most improvement seen in skin texture.

#### 2.4.2 Chest

Depending on the desired results for the depth of rhytides or scars, the number of sessions can range from one to seven with 4–12 weeks interval in between [46]. Maintained results for acne scars and rejuvenation of the skin can be seen for up to 1–2 years [39, 43]. Infections may occur, and due to HSV reactivation, prophylactic antivirals can be given a week before treatment [40]. Erythema and edema can last anywhere from 1 day to 1 week when using the Er:YAG laser [36]. However, no adverse effects were permanent and can be easily managed without affecting daily activities [36, 39, 40].

#### 2.4.3 Hands

Stebbins and Hanke performed AFL using a CO_2_ laser on 10 female patients to determine the efficacy and safety on the hands after three treatments. Although other treatment modalities have been shown to improve the appearance of pigmentation, they do not target wrinkles and texture efficiently. Patients were observed 1 month after both the first and third treatments for improvements in pigmentations, wrinkles, and texture. After both follow‐ups, improvements in signs of photoaging were seen by both the patient and the investigator. In addition, after three treatment sessions, all parameters were considerably improved compared to just one treatment. AFL is preferred over NFL due to less treatment sessions needed for the same results and minimum side effects, although AFL requires a longer healing time [35].

## 3 Sclerotherapy

Varicose veins are commonly seen on the legs due to hindered blood flow back to the heart and can run in families. However, they can also appear on the hands, face, chest, and neck [49]. Venous insufficiency leads to valvular failure, causing blood pooling and vessel damage, resulting in visible veins. Patients may seek treatment for cosmetic reasons or symptoms like superficial thrombophlebitis and leg ulcers [49].

There are three different types of sclerosant agents available: detergents, osmotic agents, and chemical irritants. Their role is to injure the vein wall and prevent recanalization by causing full thickness destruction. Examples of detergents include sodium tetradecyl sulfate (Sotradecol), polidocanol (Aethoxysclerol), sodium morruhate (Scleromate), and ethanolamine oleate (Ethamolin) [50]. Sodium tetradecyl sulfate and polidocanol are FDA‐approved; other sclerosants can be used as off‐label [51].

The minimal amount of effective concentration, indicated depending on the size of the vessel, should be used to avoid the risk of any adverse events. The needle is injected into the target vein at an angle close to the skin. Treatment of veins is done from largest to smallest [49]. Sclerosant is injected slowly until blanching develops, when resistance is felt, or when any signs of extravagation develop. Signs of extravagation may include feeling of burning pain or development of a wheal [50]. Follow‐up after 2 weeks is important to treat any areas of thrombosis, or coagula, by evacuating for faster vessel resorption and less risk of hyperpigmentation [49, 51]. If the first treatment was not sufficient, patients should wait at least 6–8 weeks for a second session (Table 5) [49].

**TABLE 5:** Sclerotherapy techniques, adverse events, and post‐procedure care on the chest and hands.

	FDA approval	Preparation	Volume needed	Method of injection	Follow‐up session	Side effects	Instructions after the injection
Sclerotherapy	Yes (Sotradecol and Asclera)						
Chest		Depending on the diameter of the reticular vein, concentrations of 0.25% (for 1–2 mm) or 0.5% (for 3 mm) STS were used [51] or 0.25% STS solution is used for veins with a diameter less than 1 mm and 0.5% for veins with a diameter of 1–3 mm [3]	0.1–2.0 mL of STS sclerosant [3]	Tessari or double‐syringe system technique, disposable 30 gauge, half‐inch needle with patients seated at a 30° angle [51]	6+ weeks [51]	No long‐term adverse events seen; post‐treatment ecchymosis lasted an average of 11.3 days. Mild pain persisted an average of 1.3 days, while edema and erythema resolved in 2–3 days [51]. Other adverse events noted in the literature for chest reticular vein treatment include telangiectatic matting, hyperpigmentation, and ulceration [3]	
Hand		0.25%, 0.5%, or 1.0% STS (Sotradecol; Bioniche Pharma, distributed by Angiodynamics Inc. Queensbury, NY) [53]	3–5 mL of sclerosant foam [53] or An average of 1.42 sessions per hand, and an average of 1.1 mL of solution per hand treatment or 5.2 mL of foam per session. The concentration of STS used ranged between 0.25% and 1.0% [53].	Tessari or double‐syringe system technique Veins were injected with foamed sclerosant from the 3 mL syringe using the direct puncture technique with a disposable 30 gauge, 1/2‐in. needle [53]		Pain, ecchymosis, edema, hyperpigmentation, erythema, and coagulum [53]	Hand was elevated (hand/ forearm parallel to the body), and massage was performed in a proximal‐to‐distal direction starting from the mid‐forearm An elastic bandage is wrapped around the mid‐forearm to hand for 24 h, and patients are told to monitor any changes in their fingers [53]

### 3.1 Neck

Sclerotherapy has not been used for cosmetic purposes for the neck in the literature.

### 3.2 Chest

Reticular and telangiectatic veins form on the chest due to a variety of origins. Reticular veins form after breast augmentation, muscle hypertrophy due to weightlifting, or from malignancies of the breast or chest wall. Telangiectasias appear due to genetic conditions, chronic photodamage, and due to increased estrogen levels. Foam sclerotherapy can be used to treat reticular veins on the chest wall. Patients are positioned at a 30° angle during treatment. Around 0.1–2.0 mL of sodium tetradecyl sulfate STS sclerosant is suitable for treatment [3]. Friedmann et al. employed a retrospective study on all patients who were treated with foam STS sclerotherapy for reticular veins on their chests: Depending on the diameter of the reticular vein, concentrations of 0.25% (for 1–2 mm) or 0.5% (for 3 mm) STS were used [51]. According to Peterson et al., 0.25% STS solution is used for veins with a diameter less than 1 mm and 0.5% for veins with a diameter of 1–3 mm [3]. In both studies, the authors stated that the detergent polidocanol can also be used to treat the reticular veins on the chest, but double the concentration would be needed due to its lower strength compared to STS [3, 51]. After contacting the patients, the results state that 7 of the 12 patients reported complete resolution, 3 reported mild to moderate improvement, and 0 patients reported no improvement.

### 3.3 Hands

Hand varicose veins can be treated cosmetically with sclerotherapy, where a detergent sclerosant is preferred over osmotic agents for its ability to migrate further in longer veins. Foam sclerotherapy, using a mix of 1 ML of 1% STS and 4 mL of air, is ideal for its safety and enhanced migration capability [52]. Treatment starts with patients seated and their hands perpendicular to their bodies. A nurse applies pressure to the forearm as a tourniquet to dilate the hand veins. Using a 0.5‐in. needle, 3–5 mL of sclerosant foam is injected into the veins, after which the grip on the forearm is released [53].

Guevara et al. studied the safety and efficacy of sclerotherapy of venous malformations on the hand and forearm. Seventeen patients were treated with either high‐concentration ethanol, STS foam, or a combination of both with an average of 2.8 mL of sclerosant. Patients were treated due to pain, swelling, or paresthesia caused by the venous malformations. Twenty‐four percent had complete resolution of symptoms, 24% had partial relief without the need for further treatment, and 35% had some improvement but required more treatment sessions. Ulceration and bleeding were seen and resolved, in 5% of the treatments, and both were seen in patients treated with ethanol. An average of 3.1 year follow‐up showed that the results are lasting [54]. Other adverse events seen with STS foam treatment include coagulum, edema, erythema, ecchymosis, pain, and very rarely hyperpigmentation [54]. In a similar study by Tremaine et al., 78.9% of patients were very satisfied, and the rest were mildly satisfied after being treated with STS foam sclerotherapy for reticular hand veins [53].

Contraindications for treatment of the hand with sclerotherapy include patients with carpal tunnel syndrome, patients with abnormal venous distribution on the dorsal hand, history of hand surgery, presence of dialysis shunts and chronic hand pain, weakness, edema, severe arthritis, or functional abnormalities [53].

## 4 Ultrasound

### 4.1 Microfocused Ultrasound

Microfocused ultrasound (MFU) is a noninvasive technique to tighten and lift the skin. The ultrasound energy is focused on a small point in the subcutaneous layers of the skin and is heated to an elevated temperature around 65°C in order to cause tissue coagulation [55]. The energy passes through more superficial layers of the skin, harmlessly, to reach the subcutaneous tissue, where it will denature the protein [55, 56]. This results in a well‐defined area of thermal injury zone surrounded by unaffected tissue. The added benefit to MFU is its capability of not affecting the tissue more superficial to the treated depth [56]. MFU can reach a depth of 5 mm in the tissue [55, 56]. When reaching the elevated temperature, collagen will begin to contract and denature; collagen will then fold and adopt a more stable configuration that is shorter and thicker. Additionally, collagen will begin to spontaneously develop in the areas of tissue coagulation, resulting in viscoelastic collagen formation [55]. The stimulation of neocollagenesis will result in skin that is more tightened and lifted [55, 56]. Indications for MFU, a noninvasive procedure, are for the skin of the face, neck, and chest to improve wrinkles (Table 6). However, other areas have been treated, including the arms, thighs, knee skin, and buttocks [56].

**TABLE 6:** MFU parameters, adverse events, and post‐procedure care on the neck and chest.

Ultrasound	FDA‐approved	Frequency, MHz	Procedural notes	Follow‐up session	Results	Adverse effects
MFU	Yes [62]					
Neck		4 MHz, 4.5 mm focal depth, 0.90 J, 350 lines *or* 7 MHz, 3.0‐mm focal depth, 0.30 J, 430 lines [59]		90 and 180 days post‐treatment [59]	According to the Physician Global Aesthetic Improvement Scale, 84% of patients experienced skin tightening improvement at the 90‐day follow‐up, while 88% showed improvement at the 180‐day follow‐up [59]	Soreness and tenderness, bruising, edema, erythema, and submandibular burns. Pain during treatment was reported as 4.1 with the 4.5 mm transducer and 2.7 with the 3.0 mm transducer, even after the use of topical anesthesia and oral analgesics [59]
Chest		10.0 MHz, 7.0 MHz, and 4.0 MHz with focal depths of 1.5 mm, 3.0 mm, and 4.5 mm, respectively [55]	280 lines of MFU‐V on three different planes. 120 lines each for 4 MHz (4.5 mm) and 7 MHz (3.0 mm) transducer, and 40 lines using the 10 MHz (1.5 mm) transducer [61]	All patients were followed up at 90 days, and only 110 patients were followed up at 180 days [61]	Aesthetic improvement: 69.9% of patients showed aesthetic improvement at the 90‐day follow‐up and 66.7% at the 180‐day follow‐up based on photographic comparisons Clinician Global Aesthetic Improvement Scale (CGAIS): 75% of patients demonstrated improvement at 90 days, with 66% showing improvement at 180 days based on live assessments. Patient satisfaction: Over 60% of patients reported satisfaction with their results at both follow‐up points, and more than 80% noticed improvements in their appearance	Pain, tenderness, bruising, pruritus, edema, soreness, paresthesia, tingling, erythema, and numbness moderate bruising [61]

MFU can be done with different energy settings in order to customize to the patient's desired result depending on the depth of the tissue that needs to be targeted [55]. Lower frequencies target deeper layers, and vice versa [56]. Therefore, the different transducers available currently can emit frequencies of 10.0, 7.0, and 4.0 MHz with focal depths of 1.5, 3.0, and 4.5 mm, respectively [55]. The different depths target the dermis at 1.5 mm, deep dermis at 3.0 mm, or the subdermal tissues at 4.5 mm, including the superficial muscular aponeurotic system (SMAS) and platysma [55, 56]. To deposit energy in more focused anatomical regions that are hard to reach with the large transducers, narrow transducers are also available with frequencies/focal depths of 10 MHz/1.5 mm and 7.0 MHz/3.0 mm [55]. Before treatment, the target coagulation zones will be mapped out for optimal healing [56].

MFU does not excite a specific chromophore like lasers do but rather targets tissue deeper than the basal skin laser, making it safe to use for darker skin tones. Improvement has been noted up to 6 months after one treatment session; therefore, it is recommended to repeat every 6–12 months [57].

Adverse effects related to MFU include discomfort and pain during the treatment, which can be reduced by pretreatment with oral NSAID, oral acetaminophen, topical anesthetics, or using the lowest energy setting that will still have results. Other adverse side effects that occur include transient erythema and edema up to 1 week. Occasional bruising, skin pigment changes, and neuropathic pain have also been reported for up to 1 month [55, 56]. Rare instances of post‐inflammatory pigmentation up to 1 month, transient numbness, muscle weakness, and striated linear skin patterns or wheals have been reported. The wheals have been reported when using 3 mm and 1.5 mm transducers, due to poor technique [55]. Results may take up to 3 months in order to be observed depending on the patient's collagen remodeling capacity, and improvement in skin laxity may last up to 10 months [58].

MFU is contraindicated on treatment zones with open wound lesions and infections (e.g, labial HSV or folliculitis), active and severe cystic acne, keloids, permanent dermal fillers, and individuals with metallic implants (e.g., pacemakers and defibrillators) [55, 58]. Patients should also avoid isotretinoin, 6 months in advance, and anticoagulation medication when being treated with MFU [58].

MFU is not recommended for older patients who have severe skin laxity, extensive photoaging, a very heavy neck, and marked platysmal banding; a better recommendation would be a surgical treatment. Therefore, MFU is preferred for younger patients with mild‐to‐moderate skin and soft tissue laxity [55].

#### 4.1.1 Neck

Lu et al. performed MFU on 25 adults, with Fitzpatrick skin types III–IV, on the face and neck area, using 7.5 and 4 MHz frequency lasers [59]. A similar retrospective study done by Fabi and Goldman on 48 women shows no association between the improvement in skin laxity and age, Fitzpatrick skin type, alcohol intake, or major illness. Patients who reported more stress, less sleep, and a BMI lower than 25 were found to have higher improvement in skin laxity, for unknown reasons [60].

#### 4.1.2 Chest

MFU became FDA‐approved for use on the chest to improve lines and wrinkles in July 2014 [55]. MFU is a favored treatment modality for treating lines and wrinkles on the chest; only a single treatment is needed for lasting results with minimal downtime. Fabi et al. performed MFU with visualization (MFU‐V) on 121 female patients. After mapping out the treatment area on the chest, each patient received at least 280 lines of MFU‐V on three different planes [61]. Each patient was graded at baseline using the Fabi/Bolton (F/B) Chest Wrinkle Scale, with 111 patients having a score of 4 (deep with well‐defined lines), and the other 15 had a score of 5 (very deep with redundant folds) [61]. When compared to the literature, the pain scores in this study were higher compared to the treatment of the face and neck with MFU‐V but similar to other chest studies and treatment of the brow and periorbital areas.

#### 4.1.3 Hands

The use of MFU on the hands has not been studied in the literature.

### 4.2 High‐Intensity Focus Ultrasound

High‐intensity focused ultrasound (HIFU) uses focused ultrasound waves to generate heat at specific depths within the skin tissue. This heat triggers a process of natural collagen production and tissue regeneration, resulting in skin tightening and lifting [63]. The superficial dermis is heated to 60°C–70°C, creating a lesion that does not damage the surrounding epidermis or other tissue [63, 64]. HIFU can be used to treat various signs of aging such as wrinkles, fine lines, and sagging skin. The procedure targets the deeper layers of the skin, including the dermis and SMAS, where it stimulates collagen production [65]. The result is improved skin texture and decreased appearance of wrinkles and fine lines. HIFU is not limited to light skin tones and can be used on darker skin tones (Table 7) [63].

**TABLE 7:** HIFU parameters, adverse events, and post‐procedure care on the neck.

Ultrasound	FDA‐approved	Frequency, MHz	Procedural notes	Follow‐up session	Adverse effects
HIFU	Yes [64]				
Neck		Platysma muscle: D4 transducer (4.5 mm, 4.0 MHz), between 1.20 and 2.00 J Deep dermis: M7 transducer (3.0 mm, 7 MHz), between 0.45 and 0.75 J Superficial dermis: S7 transducer (1.5 mm, 7.0 MHz), between 0.20 and 0.75 J [63, 65]	250–300 ultrasound impulsions [63, 65]	One session [63]	Edema, erythema, pain in the first day, and hyperpigmentation [64]

#### 4.2.1 Neck

The parameters of HIFU for use on the neck can be found in Table 7.

#### 4.2.2 Chest and Hands

The use of HIFU on the chest and hands has not been studied in the literature.

## 5 Radiofrequency

Radiofrequency (RF) is used to heat the deeper layers of the skin, including the dermis and subcutaneous tissue, to stimulate collagen production and improve the overall appearance of the skin. During RF skin rejuvenation, a handheld device is used to deliver RF energy to the targeted areas of the skin. The energy is absorbed by the skin tissue, which generates heat and stimulates collagen production. This process leads to skin tightening, improved skin texture, and reduction in the appearance of wrinkles and fine lines. RF does not target melanin; therefore it is not limited to light skin tones and can be used on darker skin tones [66, 67, 68].

There are different types of RF devices used for skin rejuvenation, including monopolar, bipolar, and fractional RF. Monopolar RF uses a single electrode with a grounding pad to deliver energy to the skin, while bipolar RF uses two electrodes without a grounding pad. In monopolar RF, the area closest to the electrode tip has the greatest concentration of electrical energy and heat, with a decrease in energy and heat as it moves further away. This type of device can cause significant discomfort; however, it also offers the deepest tissue penetration and the highest efficacy. Monopolar devices have been shown to be effective in improving skin tightening and laxity, reducing wrinkles, elevating brows, and treating both active and scarred acne lesions. Bipolar devices emit a fast and alternating current. The energy distribution is carefully regulated to minimize the discomfort that was associated with older devices. However, the main drawback is that it can only treat the volume between the two electrodes, and the depth of penetration is limited to approximately half the distance between the electrodes [66, 68].

Fractional RF delivers energy to small areas of the skin, leaving the surrounding tissue untouched, which promotes faster healing. Fractional RF uses RF energy to create tiny, evenly spaced zones of thermal injury in the skin. This is achieved by delivering RF energy through an array of small electrodes, which creates MTZs in the skin that trigger a healing response. This process stimulates the production of new collagen and elastin, resulting in improved skin texture, tone, and firmness. Fractional RF treatments are typically used to address fine lines and wrinkles, sun damage, acne scarring, and other signs of aging or skin damage (Table 8) [67].

**TABLE 8:** RF parameters, adverse events, and post‐procedure care on the neck, chest, and hands.

Laser type	FDA approval	Parameters		Procedural notes	Follow‐up session	Side effects	Avoid
		Frequency (MHz)	Consumption (W)				
RF	Yes						
Neck		Monopolar: 4 MHz [70]	120 W [70]		Two to three treatments [70]		
Chest		Multipolar, multifrequency, 1 MHz [68]		Apply pure glycerin in the beginning, move the applicator gently on the skin surface [68]	Four treatments, 2 weeks apart [68]	Some erythema and edema occurred after the treatment and disappeared within the first few hours [68]	
Hands		Monopolar: 4 MHz [69]	120 W [69]	Move device in continuous overlapping corkscrew patterns to completely cover each gel‐coated treatment zones [69]	Three treatments every 2 weeks [69]	None [69]	

### 5.1 Neck

Refer to the use of RF microneedling on the neck above.

### 5.2 Chest

Erkiert‐Polguj et al. aimed to evaluate the effect of nonablative RF rejuvenation on skin elasticity of the face and chest. The study included 20 participants who underwent four treatment sessions of nonablative RF. During the treatment, the level of energy was adjusted based on verbal feedback from the patient regarding tip warmth. Treatment was conducted once the epidermis reached a temperature of 40°C–42°C. Skin elasticity was measured using a Cutometer. The results showed a statistically significant improvement in skin elasticity 3 months after the nonablative RF treatment [68].

### 5.3 Hands

Vega et al. conducted a study across multiple centers using a monopolar RF device to treat moderate to severe hand wrinkles in 31 patients. The patients received three RF treatments at 2‐week intervals, resulting in a 50% improvement from baseline with no adverse effects. During the treatment, the level of energy was adjusted based on verbal feedback from the patient regarding tip warmth. Treatment was continued for additional 3 min when the skin surface temperature reached 40°C–42°C, and this process was repeated twice in each zone [69].

## 6 Conclusions

This comprehensive two‐part review sheds light on the diverse array of available skin rejuvenation techniques for nonfacial areas, mainly for the neck, chest, and hands. These techniques have gained popularity over the recent years with the increasing demand of nonsurgical solutions for aging. It is important to tailor the rejuvenation approach based on the patients' needs. The selection of the most suitable technique requires considering factors such as downtime and potential adverse effects; recognizing each patient's skin type, degree of aging, pain tolerance, and preference may differ. To achieve patient satisfaction, a balance between optimal results and minimal adverse effects must be met.

Grossly, the use of fillers will help addressing volume loss and skin laxity, whereas lasers can be used to manage pigment irregularities, textural concerns, and overall skin rejuvenation. The choice of filler or laser technique must remain open for discussion between the patient and practitioner. As the field continues to evolve, the emphasis on safety, efficacy, and patient satisfaction will remain fundamental, to guide the ongoing enhancement and expansion of nonfacial rejuvenation techniques.

## Conflicts of Interest

The authors declare no conflicts of interest.

## Data Availability

The data that support the findings of this study are available from the corresponding author upon reasonable request.
